# Supplementary material for: Diselenide-bond replacement of the external disulfide bond of insulin increases its oligomerization leading to sustained activity
Source: Commun Chem. 2023 Nov 21;6:258. doi: 10.1038/s42004-023-01056-4 (PMC10663622; doi:10.1038/s42004-023-01056-4)
Supplement: Supplementary file 2 — Supplementary Information [file 42004_2023_1056_MOESM2_ESM.pdf]

Diselenide-bond replacement of the external disulfide-bond of insulin increases its oligomerization leading to sustained activity

Kenta Arai[1,2*†], Masaki Okumura[3†], Young-Ho Lee[4,5,6,7†], Hidekazu Katayama[8], Kenji Mizutani[9], Yuxi Lin[4], Sam-Yong Park[9], Kaichiro Sawada[10], Masao Toyoda[10], Hironobu Hojo[11], Kenji Inaba[12,13], Michio Iwaoka[1,2*]

[†] These authors contributed equally

[*] Corresponding authors

E-mail: k-arai4470@tokai-u.jp (K. A.); miwaoka@tokai.ac.jp (M. I.)

Affiliations:

[1] Department of Chemistry, School of Science, Tokai University, Kitakaname, Hiratsuka-shi, Kanagawa 259-1292, Japan

[2] Institute of Advanced Biosciences, Tokai University, Kitakaname, Hiratsuka-shi, Kanagawa 259-1292, Japan

[3] Frontier Research Institute for Interdisciplinary Sciences, Tohoku University, 6-3, Aramakiaza Aoba, Aoba-ku, Sendai 980-8578, Japan

[4] Research Center for Bioconvergence Analysis, Korea Basic Science Institute, 162, Yeongudanji-ro, Ochang-eup, Cheongwon-gu, Cheongju-si 28119, Korea

[5] Bio-Analytical Science, University of Science and Technology, 217, Gajeong-ro, Yuseong-gu, Daejeon 34113, Korea

[6] Graduate School of Analytical Science and Technology, Chungnam National University, 99, Daehak-ro, Yuseong-gu, Daejeon 34134, Korea

[7] Research Headquarters, Korea Brain Research Institute, 61, Cheomdan-ro, Dong-gu, Daegu 41068, Korea

[8] Department of Bioengineering, School of Engineering, Tokai University, Kitakaname, Hiratsuka-shi, Kanagawa 259-1292, Japan

[9] Drug Design Laboratory, Graduate School of Medical Life Science, Yokohama City University, 1-7-29 Suehiro, Tsurumi, Yokohama 230-0045, Japan

[10] Division of Nephrology, Endocrinology and Metabolism, Department of Internal Medicine, Tokai University, School of Medicine, 143 Shimokasuya, Isehara, Kanagawa 259-1193, Japan

[11] Institute for Protein Research, Osaka University, Yamadaoka, Suita-shi, Osaka 565-0871, Japan

[12] Institute of Multidisciplinary Research for Advanced Materials, Tohoku University, Aoba-ku, Sendai 2-1-1, Japan

[13] Medical Institute of Bioregulation, Kyushu University, Fukuoka 812-8582, Japan

Table of contents

1. Supplementary Methods

1.1. General ...S3

1.2. Preparation of B chain (SeIns-B[SPys,SePys]) ...S3

1.3. Reductive unfolding of insulins ...S4

1.4. Stability assessment of insulins in human serum ...S4

2. Supplementary Figures

Supplementary Fig. 1 | HPLC chromatograms of the samples after the completion of the reaction from oxidation folding (NCA) experiments of SeIns A- and B-chain. ...S5

Supplementary Fig. 2 | HPLC chromatograms of the crude product (SeIns-B[SPys,SePys]) obtained after the deresination and purified peptide. ...S5

Supplementary Fig. 3 | HPLC chromatograms obtained from degradation experiments of BPIns ans SeIns by hIDE at pH 8.0 and 30 °C. ...S6

Supplementary Fig. 4 | HPLC chromatograms obtained from digestion experiment of SeIns-BPIns mixture by hIDE at pH 8.0 and 30 °C. ...S7

Supplementary Fig. 5 | Sedimentation profiles of SeIns, BPIns, and their mixture obtained from AUC analyses. ...S8

Supplementary Fig. 6 | Secondary structure characterization of insulins using CD spectrophotometer. ...S9

Supplementary Fig. 7 | Thermal stability of insulins in the absence of Gdn-HCl examined by CD spectrophotometer. ...S9

Supplementary Fig. 8 | Thermal stability of insulins in the presence of 1 M Gdn-HCl examined by CD spectrophotometer. ...S10

Supplementary Fig. 9 | Reductive unfolding of insulins by GSH. ...S11

Supplementary Fig. 10 | Stability assay of insulins in human serum. ...S12

3. Supplementary Table ...S13

Supplementary Table 1 | Examination of short-term toxicity of insulin analogs. ...S13

4. Supplementary References ...S14

1. Supplementary Methods

1.1. General: Bovine pancreatic insulin (BPIns) was purchased from Merck, Japan, and used after purification by reverse-phase (RP) high-performance liquid chromatography (HPLC). The HPLC conditions were same as those applied in selenoinsulin-purification (see Methods in the main text). Pooled human serum was purchased from Cosmo Bio Co., Ltd. Insulin-degrading enzyme (IDE) was purchased from Bon Opus Biosciences and used without purification. 2-Aminoethyl methanethiosulfonate (AEMTS) was synthesized according to the literature methods[1]. *N*-(9-Fluorenylmethoxycarbonyl)-*Se*-(p-methoxyphenylmethyl)selenocysteine, Fmoc-Sec(MPM)-OH, was synthesized by the literature method[2]. SeInsA and SeInsB (SeInsB[SeS]) were prepared by following our previous report[3]. Amino acid analysis (AAA) was performed using a LaChrom amino acid analyzer (Hitachi, Tokyo, Japan) after hydrolysis with a 6 M HCl solution at 150 ºC for 2 h in a vacuum-sealed tube. MALDI-TOF mass spectra were recorded using a JMS-S3000 spectrometer (JEOL, Tokyo, Japan). Plasmid used for expression of PDI was constructed as described previously. PDI was overexpressed in Escherichia coli. Cells were harvested and homogenized. Recombinant protein was purified by a combination of several types of chromatography. Purity of PDI was confirmed to be over 95% by SDS-PAGE and protein concentration was determined by BCA method [4]. All other general reagents were commercially available and used without further purification.

1.2. Preparation of SeIns-B chain (SeInsB[SPys,SePys]): The peptide elongation was performed by following the previous procedure for SeInsB[SeS][3]. Briefly, the solid phase peptide synthesis (SPPS) of the selenopeptide was carried out by a general Fmoc method from Fmoc-Ala-Wang Resin (0.89 mmol/g, 281 mg, 0.25 mmol) using Fmoc-amino acid derivative (4 equivalents)/DIC (230 μL, 1.5 mmol)-HOBt (200 mg, 1.5 mmol) or Fmoc-Sec(MPM)-OH (255 mg, 0.50 mmol)/DIC (115 μL, 0.74 mmol)-HOBt (100 mg, 0.74 mmol). Once Fmoc-Sec(MPM)-OH was introduced to a resin, solvent was changed from N-methyl-2-pyrrolidone (NMP) to dichloromethane (DCM). Completion of the couplings was assessed by the Kaiser test. After the peptide elongation, SeIns-B-resin, H-Phe-Val-Asn(Trt)-Gln(Trt)-His(Trt)-Leu-Sec(MPM)-Gly-Ser(But)-His(Trt)-Leu-Val-Glu(OBut)-Ala-Leu-Tyr(But)-Leu-Val-Cys(Trt)-Gly-Glu(OBut)-Arg(Pbf)-Gly-Phe-Phe-Tyr(But)-Thr(But)-Pro-Lys(Boc)-Ala-Wang resin (1.28 g), was obtained. A part of the resin (113 mg) was treated with TFA cocktail (TFA : 2,2'-dipyridyldisulfide : triisopropylsilane : H_2O = 90:5:2.5:2.5, 2 mL), and the mixture was vortexed for 2 h at room temperature. After the removal of TFA by N_2 stream, the crude peptide was precipitated with Et_2O, washed with Et_2O (×3) and dried in vacuo. The resulting crude peptide was purified by RP-HPLC to give SeInsB[SPys,SePys], H-Phe-Val-Asn-Gln-His-Leu-Sec(SePys)-Gly-Ser-His-Leu-Val-Glu-Ala-

Leu-Tyr-Leu-Val-Cys(SPys)-Gly-Glu-Arg-Gly-Phe-Phe-Tyr-Thr-Pro-Lys-Ala-OH (2.30 µmol, 10%). MALDI-TOF-MS (*m/z*) found: 3666.06, calcd for $[M+H]^+$: 3666.09. AAA: $Asp_{0.86}Thr_{0.98}Ser_{0.83}Glu_{2.82}Pro_{1.26}Gly_{3}Ala_{2.05}Val_{2.74}Leu_{3.96}Tyr_{2.07}Phe_{2.98}Lys_{1.32}His_{1.81}Arg_{1.07}$.

1.3. Reductive unfolding of insulins: Reductive unfolding was initiated by mixing the solutions of BPIns or SeIn (9 nmol) and GSH (90 nmol) in 100 mM Tris-HCl buffer solution (300 µL) at pH 7.5 and 25 °C. After a certain period of time, an aliquot (30 µL) was transferred into an aqueous AEMTS solution (8 mg mL^{-1}, 200 µL) chilled at 0 °C in a micro-centrifuge tube. After 15 min, the mixture was diluted with an aqueous TFA solution (0.1%, 810 µL) and stored at −30 °C. The sample solutions were analyzed by RP-HPLC by the HPLC system equipped with a sample solution loop (1 mL) and a Tosoh TSKgel ODS-100V φ 4.6 ×150 mm RP-column (Tosoh Corporation), which was equilibrated with a 80:20 (v/v) mixture of TFA (0.1%) in water (eluent A) and TFA (0.1%) in CH_3CN (eluent B) at a flow rate of 1 mL min^{-1}. After injection of the sample solution (1.0 mL) onto the HPLC system, a solvent gradient was applied: a ratio of eluent B linearly increased from 20 to 36% in 0–15 min, from 36% to 39% in 15–20min, from 39 to 40% in 20–23min. The eluted materials were detected by the absorbance at 280 nm. Peptide peak areas were integrated, and the % of remaining peptide compared to the initial was graphed against the time. The experiments were repeated 3 times for both BPIns and SeIns.

1.4. Stability assessment of insulins in human serum: Each insulin sample was dissolved in 10 mM HCl (90 nmol/100 µL), and 5.56 µL (i.e., 5 nmol of insulin) was transferred to a 1.5 mL microcentrifuge tube. The sample solution was diluted with water (19.44 µL) and then mixed with a pooled human serum (475 µL; COSMO BIO Co., LTD.), which was precentrifuged for 10 min at 13000 rpm and room temperature to remove lipid and preincubated at 37 °C for 10 min. The resulting mixture (500 µL) was incubated at 37 °C. After specific time points (0, 2, 4, 6, 10, 24, 34 h), a potion (60 µL) of the sample solution was taken up, and to the aliquot was added with cooled solution of 1% formic acid in 90% aqueous MeCN (300 µL). The resulting solution was immediately and vigorously stirred for 10 sec at room temperature by vertexing, and incubated on ice for 30 min. The suspension was centrifuged at 13,000 rpm for 10 min at room temperature. Supernatant (200 µL) was taken up and diluted with water containing 0.1 TFA (850 µL) and analyzed by RP-HPLC (Column: a Tosoh TSKgel ODS-100V φ 4.6 ×150 mm RP-column [Tosoh Corporation]). The analysis conditions were same as those described in section 1.3 in this text, except that 220 nm was used as the detection wavelength instead of 280 nm. Peptide peak areas were integrated, and the % of remaining peptide compared to the initial was graphed against the time. The experiments were repeated 3 times for both BPIns and SeIns.

2. Supplementary Figures

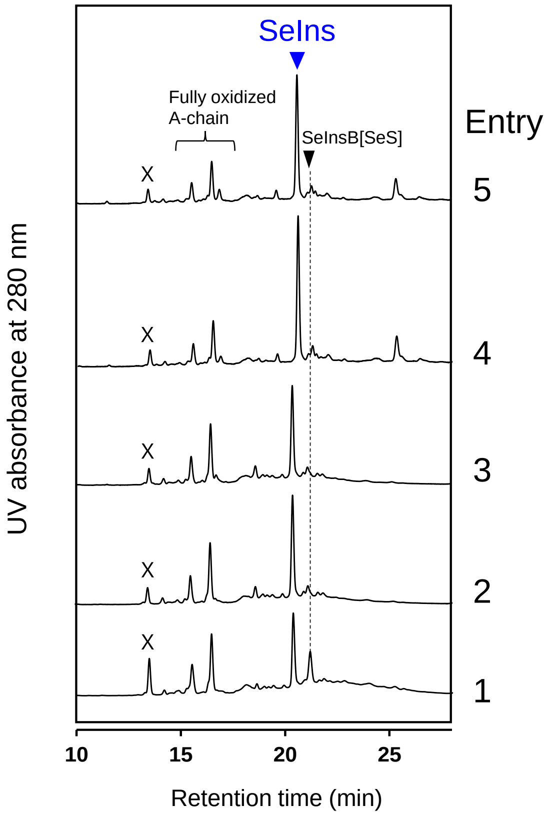

Supplementary Fig. 1 | HPLC chromatograms of the samples after the completion of the reaction from two-chain oxidative folding (NCA) experiments using separated component chains, SeIns A- and B-chain. Entry number corresponds to that in Table 1 in the main text.

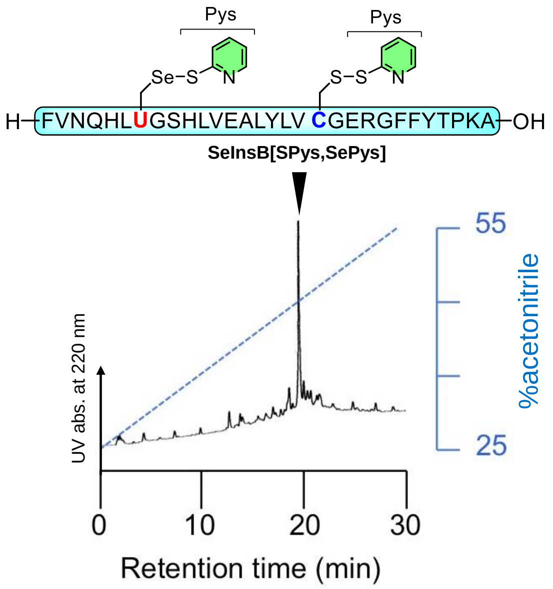

Supplementary Fig. 2 | HPLC chromatogram of a crude product obtained after the deresination of SeInsB[SPys,SePys]. The crude peptide was dissolved in aqueous 25% MeCN containing 0.1% TFA and analyzed by an HPLC system equipped a 1 mL sample loop and a MightysilRP-18 GP II (φ 4.6 mm × 150 mm) column at a flow rate of 1.0 mL/min.

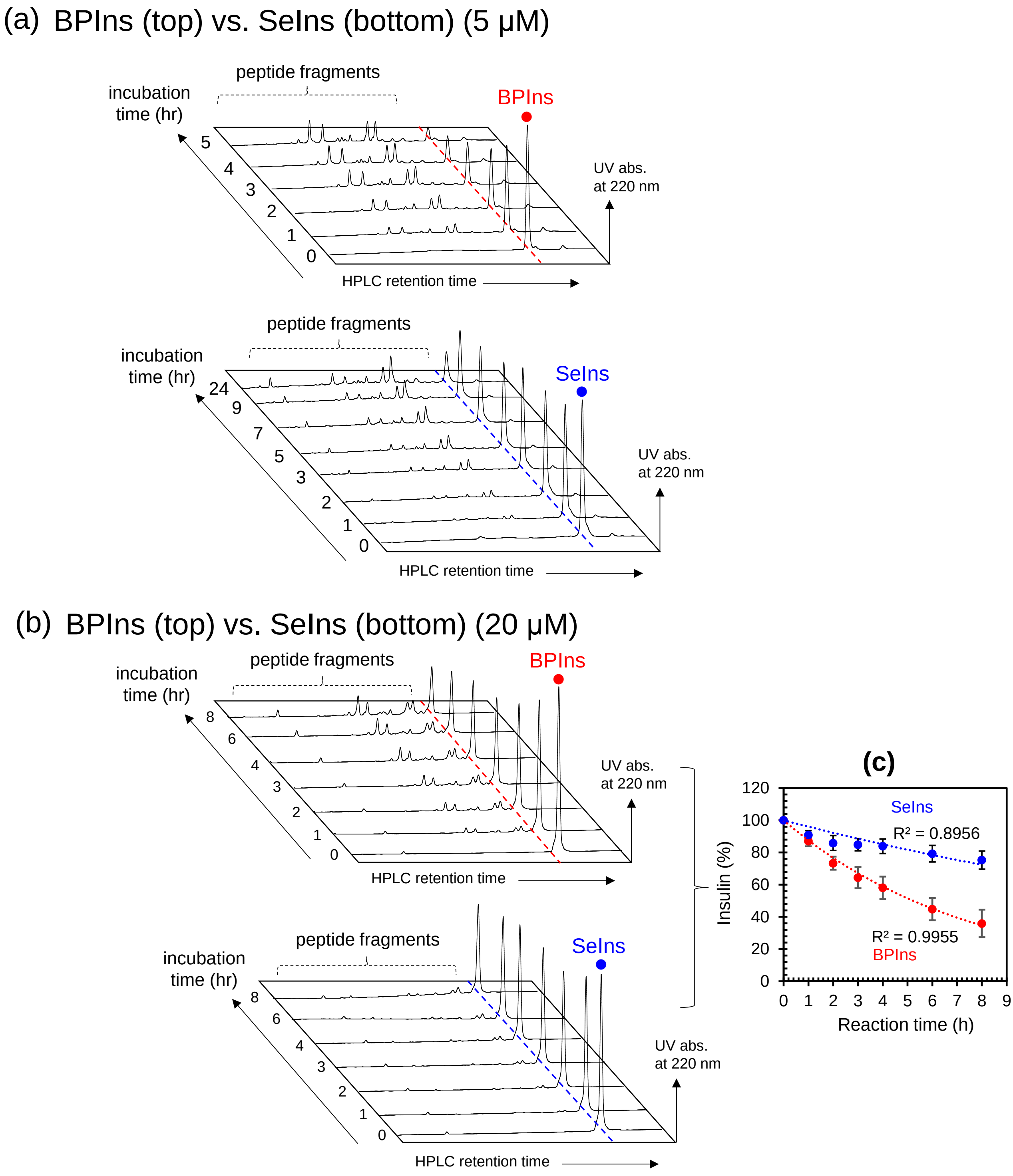

Supplementary Fig. 3 | HPLC chromatograms obtained from degradation experiments of BPIns and SeIns by IDE. (**a**) Degradation of BPIns (top) and SeIns (bottom) by IDE. BPIns or SeIns (5.0 μM) was digested by IDE (0.05 μM) at pH 8.0 and 30˚C. Decay rates of insulins observed by HPLC analyses are shown in Fig. 2b in the main text. (**b**) Degradation of BPIns (top) and SeIns (bottom) by IDE. BPIns or SeIns (20.0 μM) was digested by IDE (0.1 μM) at pH 8.0 and 30˚C. (**c**). Decay rates of insulins observed by HPLC analyses. Data are shown as mean ± SEM (n = 3)

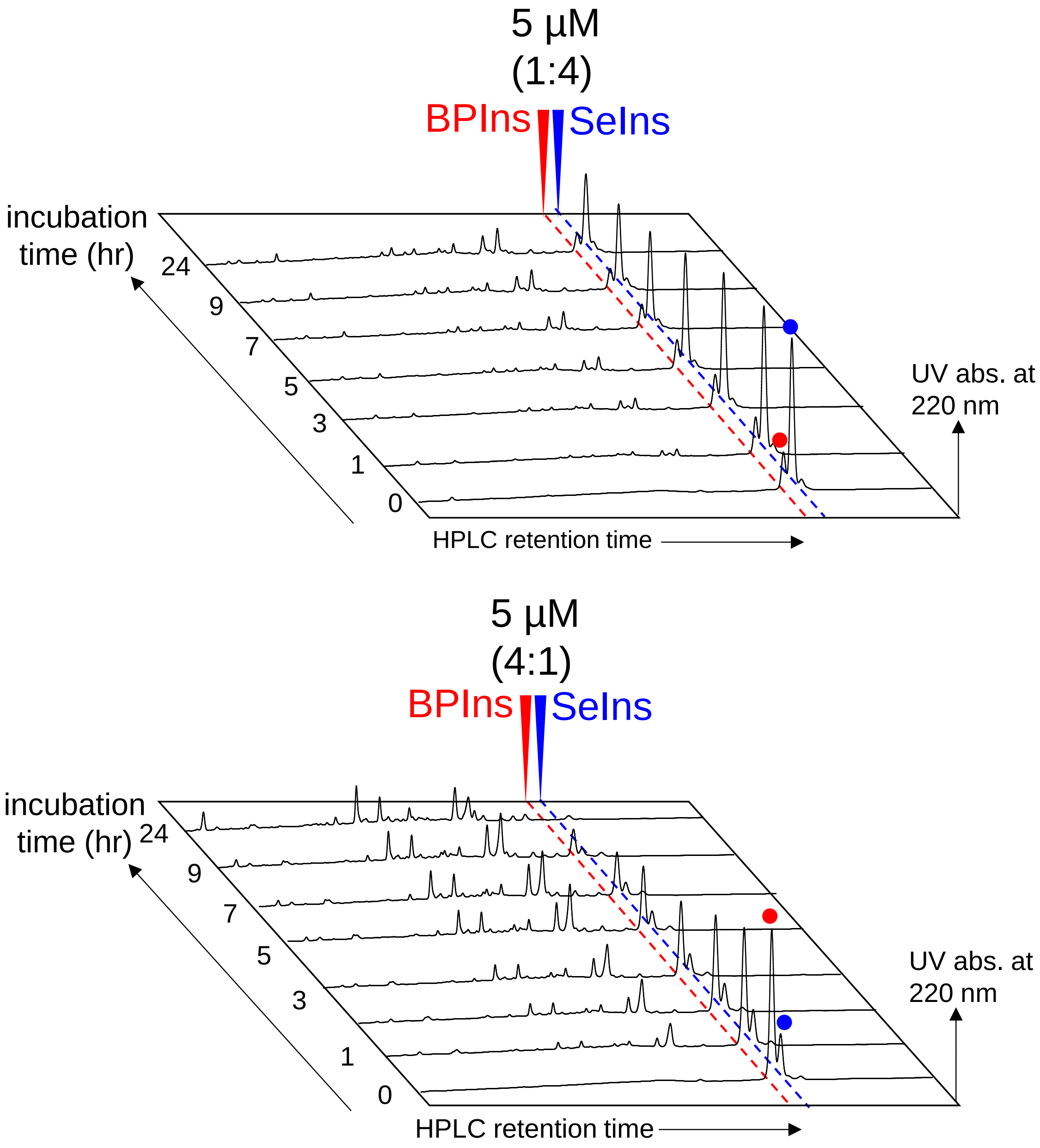

Supplementary Fig. 4 | HPLC chromatograms obtained from digestion experiments of SeIns-BPIns mixture by IDE at pH 8.0 and 30 °C. Mixture of insulins (5.0 μM; [BPIns:SeIns =1:4] in top panel, [BPIns:SeIns =4:1] in bottom panel) were incubated with IDE (50 nM) under the same conditions as those in Fig. 2b in the main text.

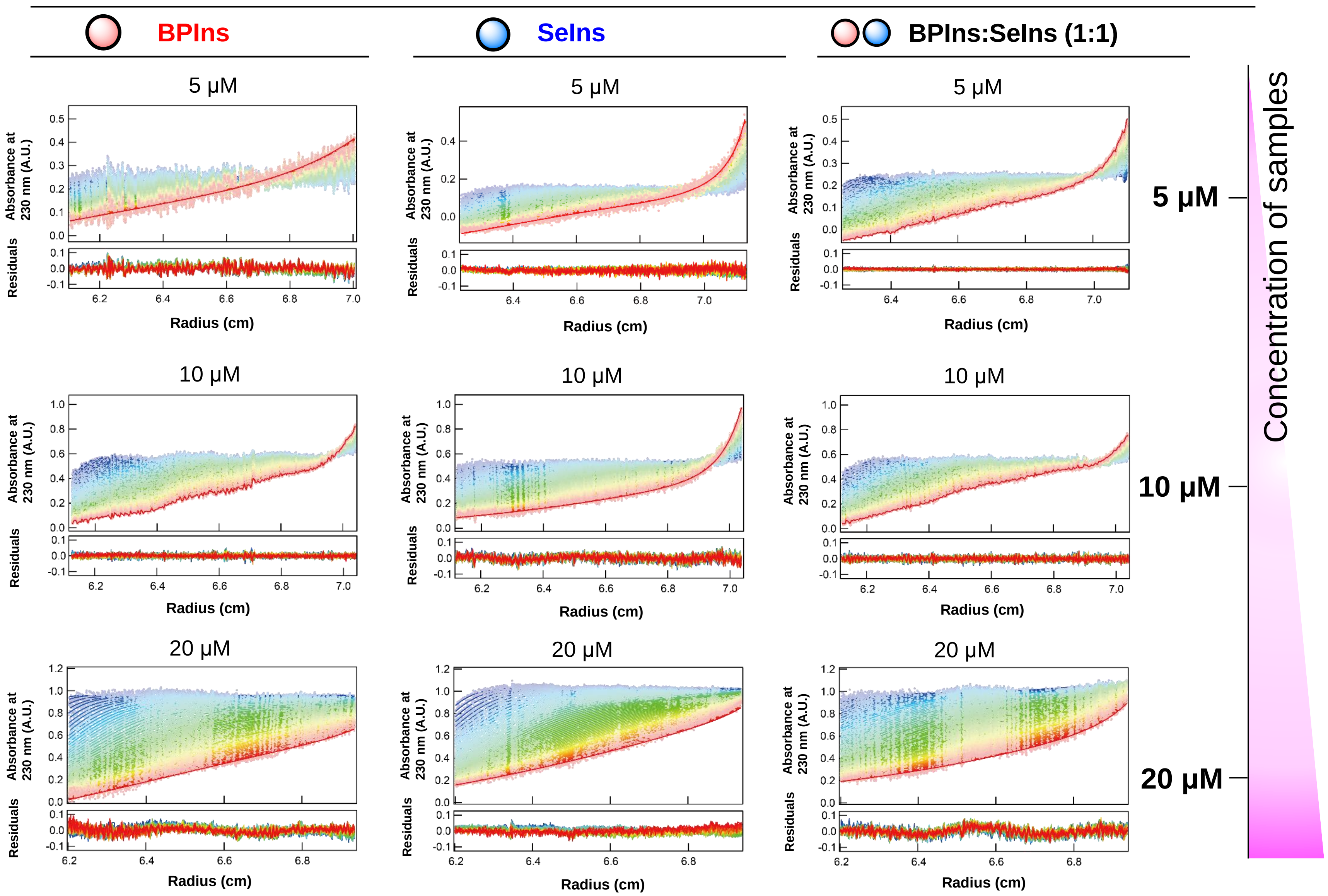

Supplementary Fig. 5 | Sedimentation profiles of BPIns (left), SeIns (middle), and their mixture (right) obtained from AUC analyses. Sample solutions were prepared at a variety of concentrations (5, 10, and 20 μM) in 0.1 M Tris-HCl buffer at pH 8.0.

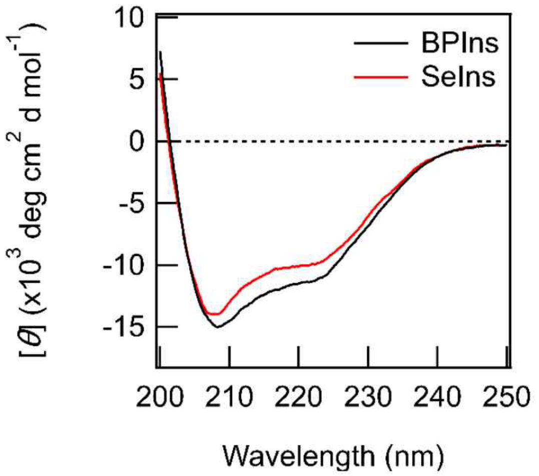

Supplementary Fig. 6 | Secondary structure characterization of insulins using CD spectrophotometer. CD spectra of SeIns and BPIns in 100 mM sodium phosphate buffer (pH 7.5) without Gdn-HCl at 25 °C. The spectra were measured at the concentration of 20 μM using a quartz cell (path length=1 mm).

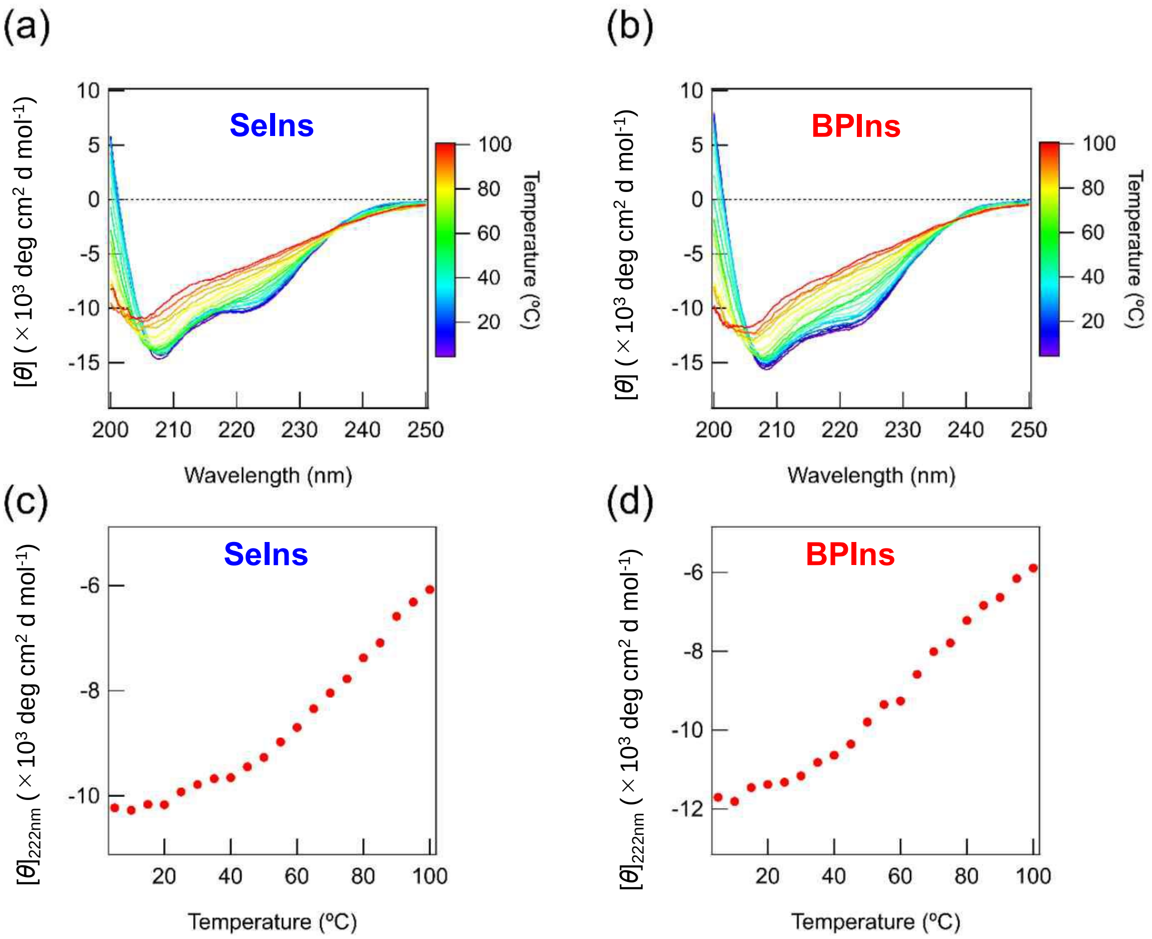

Supplementary Fig. 7 | Thermal stability of insulins without Gdn-HCl examined by CD spectrophotometer. The spectra were measured at the concentration of 20 μM using a quartz cell (path length=1 mm). (**a** and **b**) Far-UV CD spectra of SeIns (**a**) and BPIns (**b**) in the absence of Gdn-HCl from 5 to 100 °C are displayed with a color code. (**c** and **d**) Intensity changes of the CD signal at 222 nm for SeIns (**c**) and BPIns (**d**) were plotted as a function of temperature.

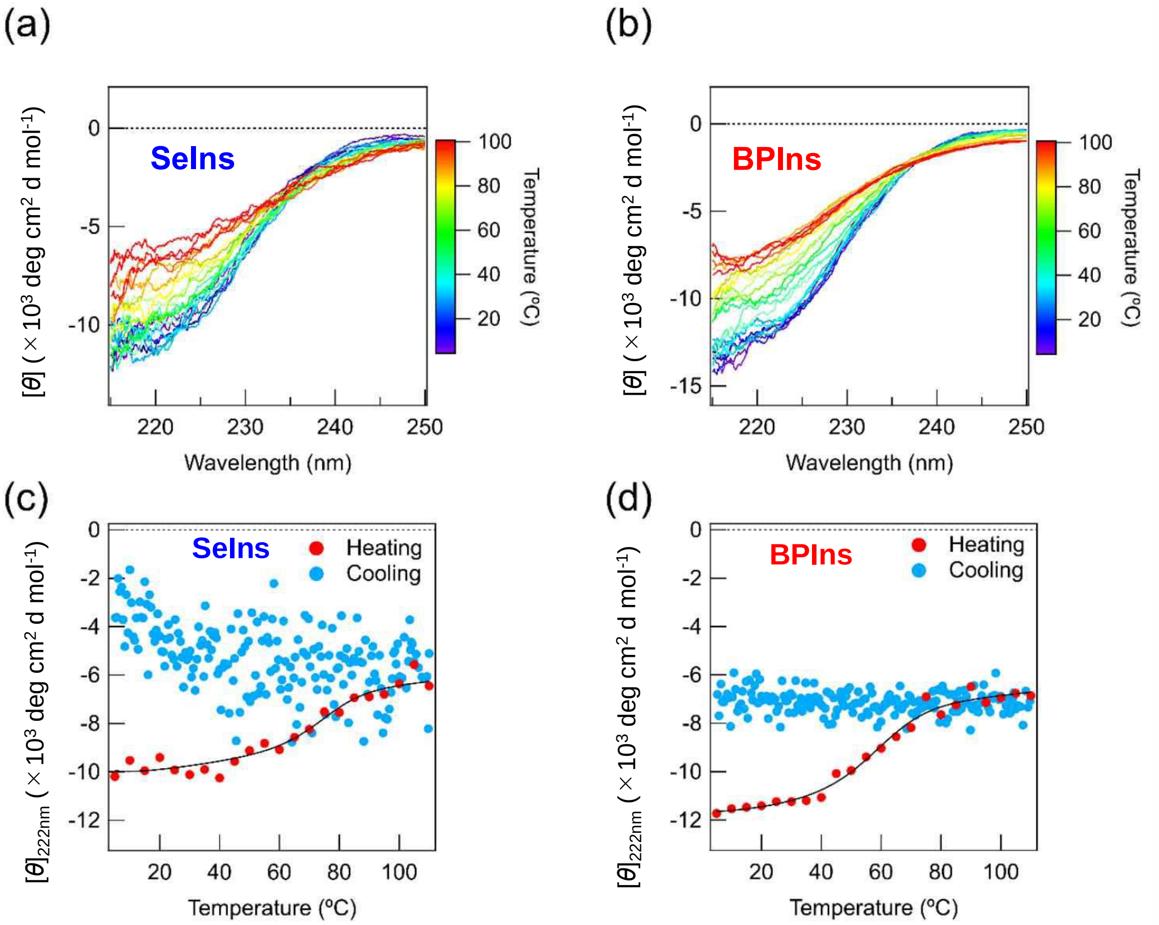

Supplementary Fig. 8 | Thermal stability of insulins with 1 M Gdn-HCl examined by CD spectrophotometer. The spectra were measured at the concentration of 20 μM using a quartz cell (path length=1 mm). (**a** and **b**) Far-UV CD spectra of SeIns (**a**) and BPIns (**b**) in the presence of 1 M Gdn-HCl from 5 to 110 °C are displayed with a color code. (**c** and **d**) Intensity changes of the CD signal at 222 nm for SeIns (**c**) and BPIns (**d**) were recorded upon heating (red) and cooling (blue). Fit curves are exhibited as a black sold line. Representative data of three independent experiments are displayed.

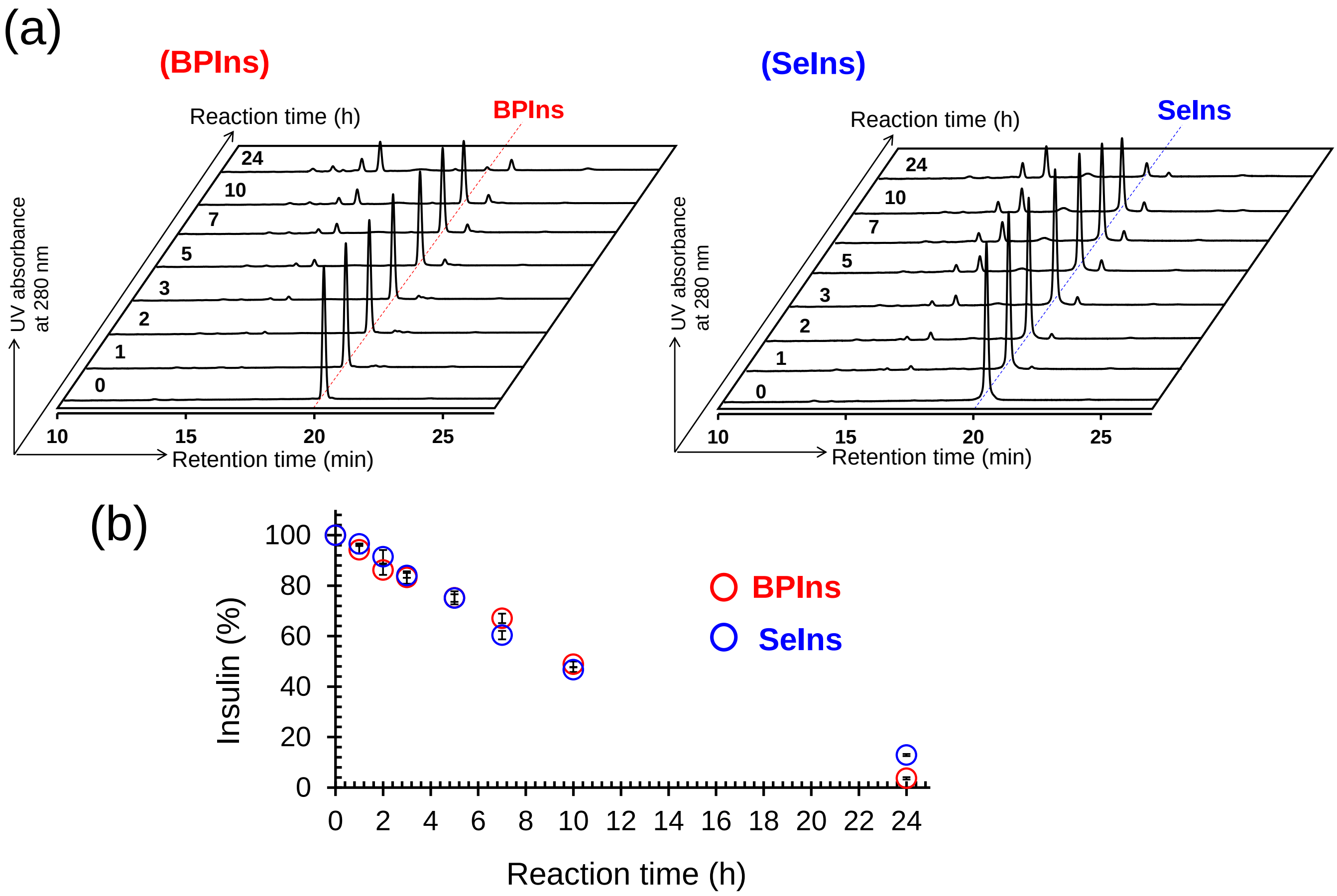

Supplementary Fig. 9 | Reductive unfolding of insulins by GSH. Reaction conditions were $[\text{insulins}]_0$ = 30 μM and $[\text{GSH}]_0$ = 0.3 mM in 100 mM Tris-HCl buffer solution at pH 7.5 and 37 °C. (**a**) HPLC chromatogram obtained from the reductive unfolding of BPIns (left panel) and SeIns (right panel). (**c**) Decay rates of insulins observed by the HPLC analyses. All data are shown as mean ± standard error of the mean (SEM) (n = 3).

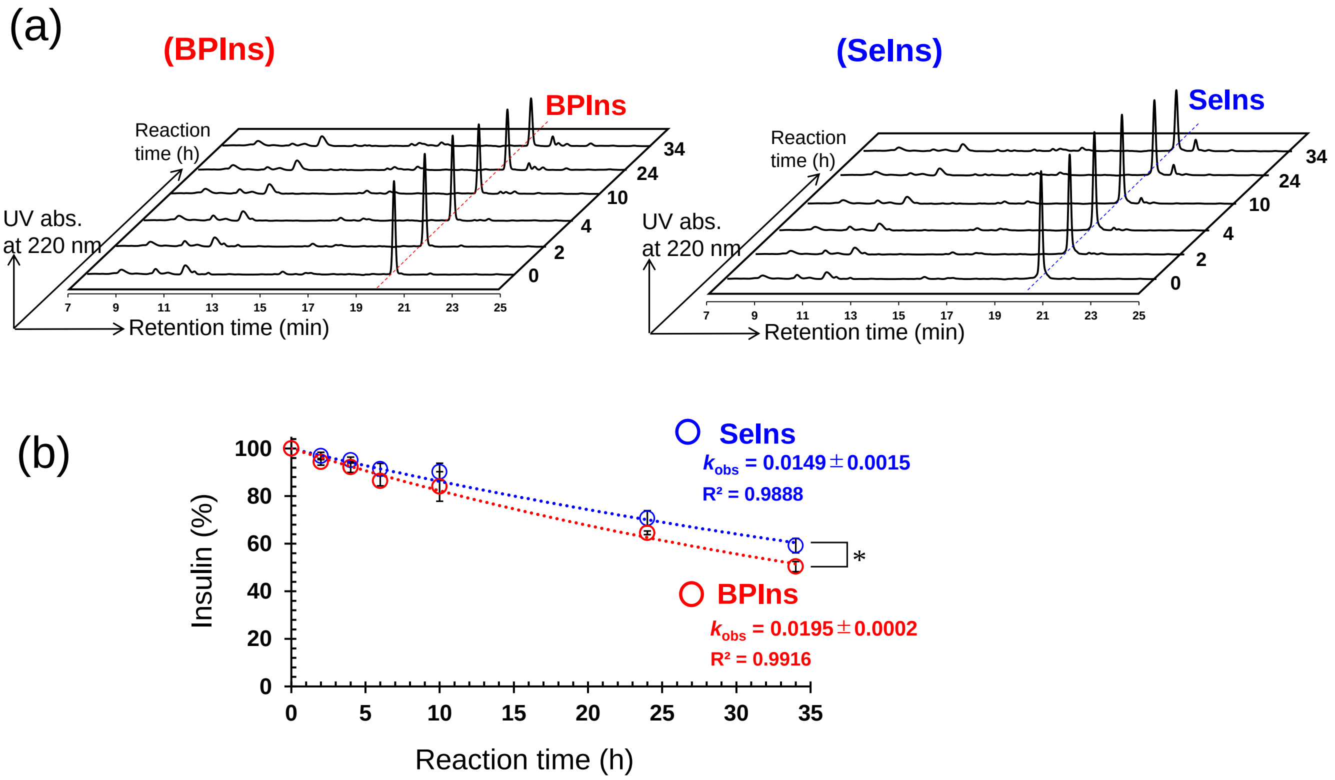

Supplementary Fig. 10 | Stability assay of insulins in human serum. Reaction conditions were [insulins]$_0$ = 10 µM in a pooled human serum at 37 °C. (**a**) HPLC chromatograms obtained from the degradation experiments of BPIns (left panel) and SeIns (right panel). (**c**) Decay rates of insulins observed by the HPLC analyses. All data are shown as mean ± standard error of the mean (SEM) (n = 3). The symbol * represents p<0.05. The p value was obtained from a t-test.

3. Supplementary Table

Supplementary Table 1 | Examination of short-term toxicity of insulin analogs.

Dosage	Insulin analog [a]	Survival rate after 24 h of single dose [%]. (Individuals surviving/total individuals)	
		**Normal rat**	**Diabetic rat**
15 μg/300 g rat	BPIns	100 (5/5)	100 (6/6)
	SeIns	100 (5/5)	100 (8/8)
150 μg/300 g rat	BPIns	100 (5/5)	100 (4/4)
	SeIns	100 (5/5)	100 (5/5)

[a] Insulin samples were prepared in saline as a solvent at concentrations of 10 units/mL and 100 units/mL for doses of 15 μg/300 g and 150 μg/300 g rats, respectively. The insulin samples were injected subcutaneously.

4. Supplementary References

1. Bruice, T. W. & Kenyon, G. L. Novel alkyl alkanethiolsulfonate sulfhydryl reagents. Modification of derivatives of L-cysteine. *J. Protein. Chem.* **1**, 47–58 (1982).
2. Shimodaira, S. & Iwaoka, M. Improved synthetic routes to the selenocysteine derivatives useful for Boc-based peptide synthesis with benzylic protection on the selenium atom. *Arkivoc* **2017**, 260–271 (2016).
3. Arai, K. *et al.* Preparation of selenoinsulin as a long-lasting insulin analogue. *Angew. Chem. Int. Ed.* **56**, 5522–5526 (2017).
4. Okumura, M. *et al.* Dynamic assembly of protein disulfide isomerase in catalysis of oxidative folding. *Nat. Chem. Biol.*, **15**, 499–509 (2019).
